# Measuring freezing of gait during daily-life: an open-source, wearable sensors approach

**DOI:** 10.1186/s12984-020-00774-3

**Published:** 2021-01-04

**Authors:** Martina Mancini, Vrutangkumar V. Shah, Samuel Stuart, Carolin Curtze, Fay B. Horak, Delaram Safarpour, John G. Nutt

**Affiliations:** 1 Department of Neurology, School of Medicine, Oregon Health & Science University, 3181 SW Sam Jackson Park Road, OP-32, Portland, OR 97239 USA; 2 Department of Sport, Exercise and Rehabilitation, Northumbria University, Newcastle upon Tyne, UK; 3 Department of Biomechanics, University of Nebraska At Omaha, 6160 University Dr S, Omaha, NE 68182 USA

**Keywords:** Wearable sensors, Freezing of gait, Parkinson’s disease, Freezing of Gait Questionnaire, Home monitoring

## Abstract

**Background:**

Although a growing number of studies focus on the measurement and detection of freezing of gait (FoG) in laboratory settings, only a few studies have attempted to measure FoG during daily life with body-worn sensors. Here, we presented a novel algorithm to detect FoG in a group of people with Parkinson’s disease (PD) in the laboratory (Study I) and extended the algorithm in a second cohort of people with PD at home during daily life (Study II).

**Methods:**

In Study I, we described of our novel FoG detection algorithm based on five inertial sensors attached to the feet, shins and lumbar region while walking in 40 participants with PD. We compared the performance of the algorithm with two expert clinical raters who scored the number of FoG episodes from video recordings of walking and turning based on duration of the episodes: very short (< 1 s), short (2–5 s), and long (> 5 s). In Study II, a different cohort of 48 people with PD (with and without FoG) wore 3 wearable sensors on their feet and lumbar region for 7 days. Our primary outcome measures for freezing were the % time spent freezing and its variability.

**Results:**

We showed moderate to good agreement in the number of FoG episodes detected in the laboratory (Study I) between clinical raters and the algorithm (if wearable sensors were placed on the feet) for short and long FoG episodes, but not for very short FoG episodes. When extending this methodology to unsupervised home monitoring (Study II), we found that percent time spent freezing and the variability of time spent freezing differentiated between people with and without FoG (p < 0.05), and that short FoG episodes account for 69% of the total FoG episodes.

**Conclusion:**

Our findings showed that objective measures of freezing in PD using inertial sensors on the feet in the laboratory are matching well with clinical scores. Although results found during daily life are promising, they need to be validated. Objective measures of FoG with wearable technology during community-living would be useful for managing this distressing feature of mobility disability in PD.

## Background

Gait disturbances, such as reduced gait speed, shorter stride length, increased time of double support and slow turns, occur early in Parkinson’s disease (PD) and progress over time [1, 2]*.* It is also estimated that over 80% of people with PD eventually develop freezing of gait (FoG), an intermittent failure to initiate or maintain locomotion [3, 4]. FoG and slow walking are the most significant factors affecting the quality of life in people with PD and are associated with an increased risk of falls [5]. FoG episodes can be very short (< 1 s), short (2–5 s) or long (> 5 s) and are more common during walking conditions typical of daily life than during straight walking in a clinic or laboratory (i.e.; turning, gait initiation, when walking through doorways or when performing a concurrent dual-task when walking [6, 7]).

Objectively assessing the severity of FoG is challenging from both a clinical and a research perspective [7, 8]. In fact, as recently summarized in our previous work [8], there still isn’t an optimal freezing score that clinicians can use. The ‘gold-standard’ to assess the presence of freezing (from actual video recordings [9, 10] or computer-generated animations [11]) is time consuming and does not represent daily fluctuations. Assessment of FoG in the clinic or laboratory is challenged by the fact that these assessment do not accurately represent severity or extent of FoG in daily life [12, 13]. Increased attention, alertness, and effort to impress the examiner during testing may improve gait performance [14–16]. This is particularly true for FoG, in fact FoG is difficult to elicit during a clinical visit or in the laboratory [13, 17, 18] when participants focus attention on their walking. As walking and turning while dual‐tasking (DT) have been suggested to induce freezing, the addition of a DT is often used to elicit FoG in the laboratory environment [12, 19].

Significant advancements in technology using wearable inertial sensors provides a new opportunity to objectively quantify subtle gait disturbances, such as FoG, in both clinical and laboratory settings [20–22], and ultimately during daily life [20, 21]. Objective measures of gait disturbances, such as FoG, have the potential to help inform effects of treatment, disease progression, and characterize fall risk.

Two recent reviews [23, 24] have summarized different approaches to objectively measure FoG with wearable sensors. However, only three studies were performed in the home setting and the validity of the algorithms in the laboratory or home varied considerably (accuracy 79% to 96%) [25–28]. While studies detecting FoG in a laboratory setting have been well-validated, studies focused on detecting FoG during daily life are relatively scarse [23, 25, 26, 29–32]. In addition, the percentage of freezing during daily life, as well as the variability of it, have not yet been reported. In addition, the impact of FoG on mobility perception and other gait disturbances have not yet been investigated during daily life. Finally, open-source solutions to monitor FoG with wearable inertial sensors in free living conditions are not yet available. Common algorithms available to many investigators will improve reproducibility of results, external validation, algorithm improvements, and ultimately reducing barriers to applying digital health solutions for unsupervised FoG monitoring.

Here, we aimed to: (1) introduce a novel, objective algorithm to detect FoG episodes in the laboratory in a cohort of people with PD with and without freezing of gait compared to age-matched controls, and evaluate the performance of such algorithm with clinicians judgment of FoG; and (2) extend this approach to characterize FoG during daily life (7 days recording with inertial sensors on the feet) as well as investigate the association between subjects’ perception of freezing severity and other objective measures of walking and turning in a different cohort of people with PD with, and without, freezing of gait.

## Methods

We analyzed the dataset of two studies: Study I took place in the laboratory to determine validity of detected freezing events compared to expert rating of videos and Study II took place in the home setting to compare FoG episodes and gait between those with, and without, FoG.

## Participants

Study I included 45 subjects with PD and 21 healthy controls of similar age while Study II included 48 subjects with PD. All participants were recruited through the Parkinson’s Center of Oregon clinic at Oregon Health & Science University. For both studies, inclusion criteria were: diagnosis of idiopathic Parkinson’s disease confirmed by a movement disorders neurologist according to the United Kingdom Parkinson’s disease Society Brain Bank criteria, Hoehn and Yahr scores of II–IV, and ability to follow instructions and appreciate research purpose. For both studies, exclusion criteria were: other factors affecting gait (hip replacement, musculoskeletal disorder, uncorrected vision or vestibular problem), or an inability to stand or walk without an assistive device. Study I included 27 participants classified as freezers, based on a score of > 0 on the New Freezing of Gait Questionnaire (NFOGQ) [33] and 18 without FoG. They were all tested in our laboratory in the “Off” state, after at least 12-h overnight withdrawal from anti-parkinsonian medications. We choose to test people with PD Off their medication because FoG is most often observed in Off periods [34]. Study II included 23 different participants with PD classified as freezers, based on a score of > 0 on the NFOG Q as well as 25 participants with PD without FoG.

Both studies were carried out in accordance with the recommendations of the Oregon Health & Science University (OHSU) institutional review board (IRB) with written informed consent from all subjects. All subjects gave written informed consent in accordance with the Declaration of Helsinki. The protocols were approved by the OHSU IRB (#9903, #10775 and #15578).

## Procedure

### Procedure for study I

Participants underwent a 3-h assessment, which included clinical assessments, questionnaires, and quantitative assessments of gait, detailed elsewhere [35]. The gait assessment analyzed here included two walking conditions, a 2-min walk in a 8-m hallway, and a 1-min walk with a concurrent cognitive task (reciting alternate letter of the alphabet). Participants were asked to stand quietly for few seconds, instructed when to walk 8-m at their comfortable speed, turn 180°, and keep walking until they hear stop at the end of 2 (or 1) min test. While performing these walking tasks, participants wore eight wireless, synchronized inertial sensors (Opals by APDM, Inc.) on both shins, feet, wrists, on the sternum and on the posterior trunk (over L5). Each inertial sensor includes a tri-axial accelerometer, gyroscope, and magnetometer sampling at 128 Hz. Data were wirelessly streamed to a laptop and stored for offline analysis. All trials were video-recorded and videos were rated by two movement disorders specialist (DS and JGN), blinded to group allocation, who assessed each FoG episode, its duration (short, medium or long) and and total number of FoG episodes per test. Disease severity was measured with the Movement Disorders Society Unified Parkinson’s Disease Rating Scale (MDS-UPDRS) Part III [36]. The MDS-UPDRS Part III was administered by a certified examiner.

### Procedure for study II

Subjects wore three inertial sensors (same as Study I) only one on each foot and one over the lumbar area for a week of continuous monitoring for at least 8 h/day, details in Shah et al. [37]. The Opal is lightweight (22 g), has a battery life of 16 h, and includes 8 GB of storage, which can record over 30 days of data. Subjects removed the sensors at night and placed them in a charging station. Data were stored in the internal memory of the Opals. Subjects returned the sensors either by mail, using a pre-paid box after completion of 1 week of data collection, or a research assistant picked up the sensors at their homes. Data were uploaded via a laptop to a cloud service and downloaded to a local computer for analyzing FoG. Severity of disease was rated based on MDS-UPDRS, Part III, while participants were on their regular dose of Levodopa (On state, approximately 1 h after intake). The MDS-UPDRS Part III was administered by a certified examiner in a laboratory screening visit. Perceived motor functioning was assessed using the mobility domain of the Parkinson's Disease Questionnaire (PDQ-39) [38]. The PDQ-39 mobility Sect. (10 items) has a possible score range of 0 to 40; higher scores are associated with greater impairment.

## Data analysis

### Video assessment of freezing (study I)

Two independent raters, both with experience in FoG assessment, analyzed the video-recordings. A FoG episode was defined when the gait pattern (alternating right and left steps) was arrested or if it appeared as if they were trying unsuccessfully to initiate or continue locomotion/turn. The end of an episode was defined as the time when an effective step had been performed and followed by continuous locomotion. At least two effective steps were required in order to score and time the duration of a freezing episode. The raters were asked to sum, for each subject, and for each gait condition (walking in single- and dual-task conditions) the number of FoG episodes using a similar cut‐off duration as in the NFOGQ: less than 1 s (very short episodes), 2–5 s (short episodes), and more than 5 s (long episodes).

### Freezing detection algorithm, open-source (study I and study II)

**Fig. 1:**
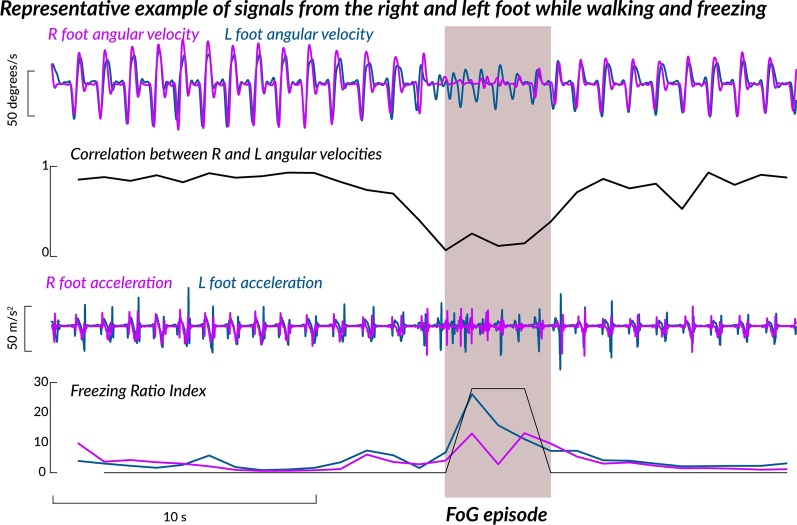
Representative example of signals while walking and freezing

**Fig. 2:**
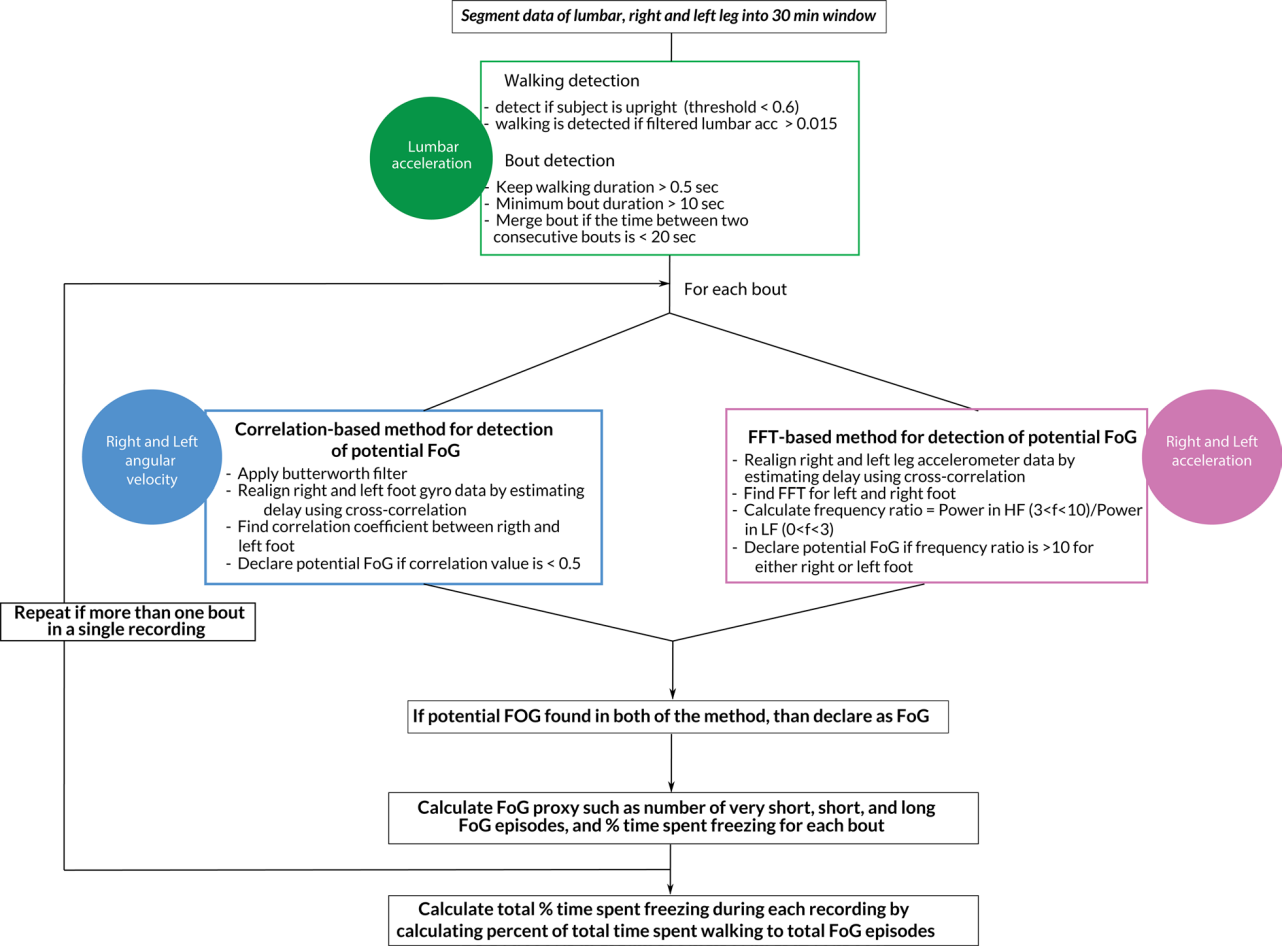
Schematic of the algorithm for FoG detection

We present an open-source algorithm to detect numbers of FoG episodes, percentage of time spent freezing and its variability during 7 days of unsupervised, daily life settings (Figs. 1 and 2). We first used the proposed algorithm to compare the number of detected FoG episodes with the clinical judgment of two movement disorders experts using Study I, and then apply the proposed algorithm on Study II to characterize FoG during 7 days of unsupervised monitoring in daily life.

The algorithm first detects the periods of walking, from the 3D angular velocity and 3D acceleration of the lumbar sensor, in windows of 30 min [39–42]. Briefly, walking bouts of 10 s and longer were then used for further analysis. Although the algorithm was originally written for sensors to be placed on the feet, in Study I we also compared the performance of the algorithm using sensors on the shins.

The FoG algorithm used the antero-posterior accelerations and rotations around the medio-lateral axis of the gyroscopes of the sensors worn on the feet during each identified gait bout to identify potential FoG episodes. It has been shown that freezing is usually accompanied by high-frequency leg movements [4]. Recently a ‘Freezing Ratio’ was defined as the power in the “freeze band” (3.5–8 Hz) divided by the power in the “locomotor band” (0.5–3 Hz) with larger ratios indicating more freezing [43, 44]. Such ‘high-frequency’ components of gait have been associated with the ‘trembling’ observed during freezing episodes. In addition, it has been recently shown that during regular walking, the correlation between right and left foot angular velocity is high, while it drops significantly prior to, and during, a FoG episode [45, 46]. Adopting a similar idea, we used here the information from both accelerometers and gyroscopes during non-overlapping windows of 1 s to confirm a FoG episode.

The flowchart of the algorithm is shown in Fig. 2. First, we use data from the medio-lateral gyroscope to determine the correlation between left and right leg, and if the correlation value goes below 0.5 than declare that 1-s window as potential FoG episode. Second, we use antero-posterior accelerometer data to calculate a freezing ratio defined as the ratio of the energy of the signal during 3–10 Hz (high frequency) to energy of the signal during 0–3 Hz (low frequency) using FFT. If the freezing ratio is higher than 10, then, that period of time is declared as a potential FoG episode. Finally, we declare an actual *FoG episode* if a specific 1-s window is declared as potential FoG by both methods (See Fig. 1). Once FoG episodes are identified, we grouped them into three categories identified in the NFOGQ: very short FoG (< 1 s) episodes, short FoG (2–5 s) episodes, long FoG (> 5 s) episodes. Percentage time freezing was calculated as percentage of total time spent freezing over total time spent walking in 30 min windows. Our primary outcome measure for freezing was the cumulative sum of such percentages across a week of daily recording. In addition, the variability of the percentage of time spent freezing was reported.

Initially, we did not merge any individual freezing episodes detected by the algorithm, and the data set (Study I) was analyzed and compared with data obtained from clinical raters. While tuning the algorithm, we realized that the algorithm may split long freezing episodes into multiple, small freezing episodes. This phenomenon could be explained by hesitations or steps during freezing, which woube be detected by the algorithm (and identified as non-freezing episodes) but maybe not by the clinical eye. Therefore, we tried various thresholds to decide whether to split or not a FoG episode and compared results obtained with the clinicians scores. Results are in Appendix Table 5 and lead to the choice of merging freezing episodes with hesitation durations ≤ 2 s as one, single, freezing episode.

**Table 1:** Demographics and clinical characteristics for participants in Study I and Study II

Study I	Non-freezers (N = 18)		Freezers (N = 27)		p-value
	Mean	SD	Mean	SD	
Age (years)	70.3	7.0	69.6	7.4	0.723
Disease duration (years)	8.2	4.7	9.0	6.3	0.862^a^
UPDRS III *ON*	43.6	11.6	46.7	12.2	0.402
Gender (F/M)	4/14		6/21		1

Study II	Non-freezers (N = 25)		Freezers (N = 23)		p-value
	Mean	SD	Mean	SD	
Age (years)	67.8	4.8	69.6	7.1	0.320
Disease Duration (years)	6.6	3.7	13.0	6.5	**0.0002**
UPDRS III *ON*	33.7	9.9	34.1	12.7	0.913
Gender (F/M)	14/11		6/17		0.071

^a^Mann-Whitney U-test

### Turning and walking features (study II)

In addition to FoG characterization, we report the following turning and walking features during daily life monitoring (Study II), using methodology published elsewhere [37, 47], not part of the open-source algorithm.

*Turning features* the algorithm used the horizontal rotational rate (yaw) of the lumbar sensor during each identified gait bout. Details are described elsewhere [39]. Briefly, a turn was defined as a trunk rotation around the vertical plane with a minimum of 15 °/s [39]. Turning angle was then obtained integrating the angular rate of the lumbar sensor around the vertical axis (done separately for each turn). The following turning characteristics were averaged across the week for number of turns per each 30-min period: number of turns, average turning angle (°), average turning duration (s), average turn peak velocity (°/s) and the coefficient of variation (CV) was calculated for the turn peak velocity.

*Walking features* A separate algorithm, using Unscented Kalman Filter to fuse information from the accelerometers, gyroscopes, and magnetometers to estimate the orientation and position trajectory of the sensors on the feet, was used to quantify quality of walking [37, 47]. Gait was defined as walking bouts of at least 3 consecutive strides, a minimum duration of 3 s and intermittent breaks of no longer than 6 s. The selected outcomes for walking were gait speed (m/s), and the pitch angle of the foot at initial contact (°) selected as an indicator of shuffling as well as the variability of the pitch angle of the foot at initial contact. All outcomes were averaged across the hours of recording. Steps that occurred during turning were excluded.

### Statistical analysis

In both studies, Independent sample *t*-tests compared age, disease duration, and MDS-UPDRS Part III between people with and without FoG.

*Study I* To evaluate the agreement between the two clinical raters, and between the algorithm and the raters, we used an Intra-Class Correlation Coefficient (ICC) [48]. In accordance with previous studies [11, 49], we used the following classification of ICC power: < 0.2 negligible, 0.2 ≤ 0.4 weak, 0.4 ≤ 0.7 moderate, 0.7 ≤ 0.9 strong, and > 0.9 very strong. In addition, the mean and 95% ICC confidence interval as well as the number of FOG events were reported. To investigate the performance of the algorithm against the classification of freezer vs. non-freezer based on clinical raters, we computed the Area Under Curve (AUC), and various performance metrics (such as accuracy, sensitivity, specificity).

*Study II* To investigate whether quantity and quality of mobility outcomes differed between the two groups, linear mixed models were fit for each outcome, with and without adding disease duration as a covariate to account for variations in the presented outcomes with disease duration. Lastly, Pearson’s correlation was used to evaluate the association between objective measures of freezing and the clinical or extracted measures of quantity and quality of mobility at home. The statistical analysis was performed in MATLAB R2018b (The Mathworks Inc., Natick, MA, USA) using the Statistics and Machine Learning Toolbox. A significance level of 0.05 was used throughout.

## Results

Briefly, people with and without FoG were similar in both studies for age, and MDS-UPDRS III, while people with FoG presented higher disease duration compared to people without FoG (p < 0.05), only for the participants of Study II. Demographics and clinical characteristics of participants with PD for Study I and II are summarized in Table 1.

### Study I: comparison between objective and clinically detected FoG

A total of 79 FoG events were identified from clinician rater I and a total of 150 FoG episodes were identified from clinician rater II. The discrepancy between the two raters was mainly due to the detection of the very short FoG episodes (< 1 s) with an ICC of 0.39, while the agreement is overall strong for short (2 to 5 s) and long episodes (> 5 s) with ICC of respectively 0.839 and 0.875. In general, ICCs during dual-task walking showed a tendency to be lower compared to ICCs calculated for single-task walking for very short and short FoG episodes. Table 2 shows the number of identified episodes and ICCs for single- and dual-task walking separately as well as overall between raters and between raters (averaged) and the algorithm with sensors in two different placements (feet and shins). The overall ICCs were higher between the objective FoG calculated from the sensors on feet and the average clinical raters compared to the ICCs between the objective measures of FoG calculated from the sensors on the shins and the average clinical raters. In addition, the ICCs for the very short FoG episodes were poor for sensors on the feet or shins.

**Table 2:** ICC between the two clinical raters, as well as ICC between the average of the clinical raters and the algorithm based on IMUs on the feet and the algorithm based on IMUs on the shins

Video	# of detected episodes		ICC (2,1)- CI	# of detected episodes		ICC (2,1)- CI	# of detected episodes		ICC (2,1)- CI
Rater 1 vs Rater 2	Rater 1	Rater 2	Very Short episodes (< 1 s)	Rater 1	Rater 2	Short episodes (2 to 5 s)	Rater 1	Rater 2	Long episodes (> 5 s)
Walk 2-min ST	7	45	0.396 (− 0.210 to 0.698)	35	31	0.892 (0.795 to 0.943)	13	9	0.848 (0.707 to 0.921)
Walk 1-min DT	3	25	0.346 (− 0.375 to 0.689)	13	30	0.743 (0.492 to 0.870)	8	10	0.929 (0.857 to 0.964)
Overall	10	70	0.390 (− 0.005 to 0.629)	48	61	0.839 (0.746 to 0.899)	21	19	0.875 (0.801 to 0.922)

Wearable sensors on feet	# of detected episodes		ICC (2,1)- CI	# of detected episodes		ICC (2,1)- CI	# of detected episodes		ICC (2,1)- CI
Algorithm vs Raters	Algorithm	Raters	Very Short episodes (< 1 s)	Algorithm	Raters	Short episodes (2 to 5 s)	Algorithm	Raters	Long episodes (> 5 s)
Walk 2-min ST	71	26	0.474 (0.025 to 0.718)	36	33	0.915 (0.841 to 0.955)	7	11	0.872 (0.761 to 0.932)
Walk 1-min DT	57	14	0.355 (− 0.161 to 0.658)	12	22	0.387 (− 0.171 to 0.683)	6	9	0.932 (0.865 to 0.966)
Overall	128	40	0.431 (0.018 to 0.662)	48	55	0.818 (0.714 to 0.884)	13	20	0.895 (0.833 to 0.934)

Wearable sensors on shins	# of detected episodes		ICC (2,1)- CI	# of detected episodes		ICC (2,1)- CI	# of detected episodes		ICC (2,1)- CI
Algorithm vs Raters	Algorithm	Raters	Very Short episodes (< 1 s)	Algorithm	Raters	Short episodes (2 to 5 s)	Algorithm	Raters	Long episodes (> 5 s)
Walk 2-min ST	94	26	0.374 (− 0.175 to 0.666)	24	33	0.703 (0.443 to 0.842)	4	11	0.688 (0.414 to 0.833)
Walk 1-min DT	49	14	− 0.176 (− 1.306 to 0.400)	20	22	0.619 (0.252 to 0.806)	4	9	0.814 (0.636 to 0.905)
Overall	143	40	0.304 (− 0.095 to 0.558)	44	55	0.672 (0.485 to 0.792)	8	20	0.744 (0.597 to 0.837)

**Fig. 3:**
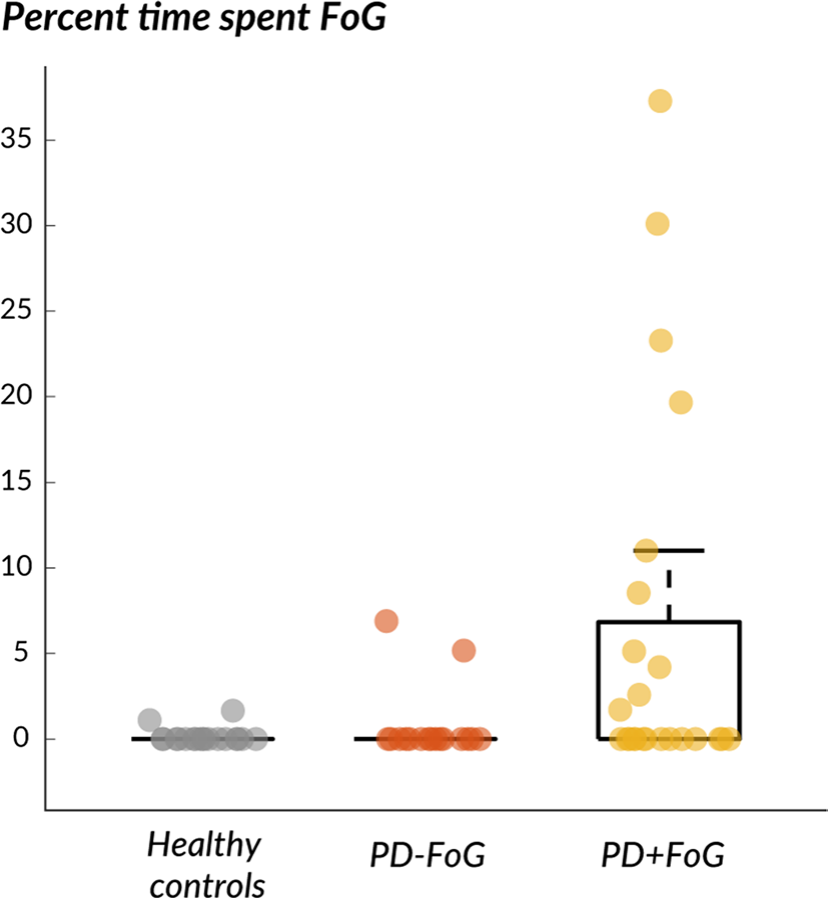
Box plot of the percent of time spent freezing during walking in the laboratory in healthy controls, PD − FoG and PD + FoG

Moreover, healthy controls and PD-FoG showed a significantly lower % time spent freezing during walking compared to PD + FoG, see boxplot in Fig. 3.

The algorithm performance to classify non-freezers vs. freezers against the clinical raters are summarized in Table 3. Specifically, the AUC value to discriminate non-freezers from freezers using the algorithm versus clinical rater 1 was 0.93, and versus clinical rater 2 was 0.89. The performance of the algorithm were slightly higher when compared to clinical rater 1 compared to clinical rater 2.

**Table 3:** Performance of the algorithm with clinical raters in classifying Non-freezers vs Freezers

Non-freezers vs freezers	AUC	Best Threshold	Accuracy	Sensitivity	Specificity	False positive rate	False negative rate
Rater 1 vs algorithm	0.93	0.09	0.88	0.89	0.88	0.13	0.11
Rater 2 vs algorithm	0.89	0.13	0.85	0.80	0.87	0.13	0.20

### Study II: FOG in daily life

Based on the ICC results of Study I, we deemed it unreliable to record very short FoG episodes during daily life. Therefore, Study II used the percent of time spent FoG as a sum of short (2 to 5 s) and long (> 5 s) FoG episodes. The extracted FoG proxy, percent of time spent FoG (average of 30-min windows) was higher (p = 0.04) while the variability of time spent FoG was lower (p = 0.02) in people who identified themselves as freezers, according to the NFOG, compared to people who identified themselves as non-freezers, see details in Table 4. After adding disease duration as a covariate, such differences between freezers and nonfreezers did not change (p < 0.01).

**Table 4:** Means and SD of the quantity and quality of mobility measures over 7 days of continuous monitoring

Quantity of mobility	Non-freezers		Freezers		p-value	p-value (adjusted)
	Mean	SD	Mean	SD		
Total recording (hours)	49.7	9.3	52.0	19.4	0.600	0.855
Average turns #	38.3	15.3	45.2	28.2	0.300	0.827
Average bouts #	6.2	1.0	6.3	1.4	0.680	0.312
Total bouts time (hours)	3	1.5	4.2	3.1	0.08	0.22
Freezing proxy						
Time spent freezing (%)	15.42	4.69	20.18	10.15	**0.040**	**0.006**
CV time spent freezing (−)	0.848	0.224	0.703	0.206	**0.024**	**0.007**
Quality of mobility						
Average gait speed (m/s)	0.87	0.13	0.87	0.24	0.960	0.802
Average pitch angle (°)	− 17.0	5.1	− 13.1	6.0	**0.020**	0.108
CV Pitch Angle (−)	− 0.487	0.238	− 0.746	0.415	**0.010**	0.154
Average turn angle (°)	94.3	4.6	87.2	4.9	** < 0.0001**	** < 0.0001**
Average turn duration (s)	2.1	0.2	1.9	0.3	**0.001**	0.056
Average turn peak velocity (°/s)	77.0	9.4	80.5	12.4	0.270	0.924
CV turn peak velocity (−)	0.311	0.031	0.311	0.032	0.940	0.744

p-values from linear mixed models are reported, considering (p-value adjusted) or not (p-value) disease duration as covariate, p-values < 0.05 are in bold

**Fig. 4:**
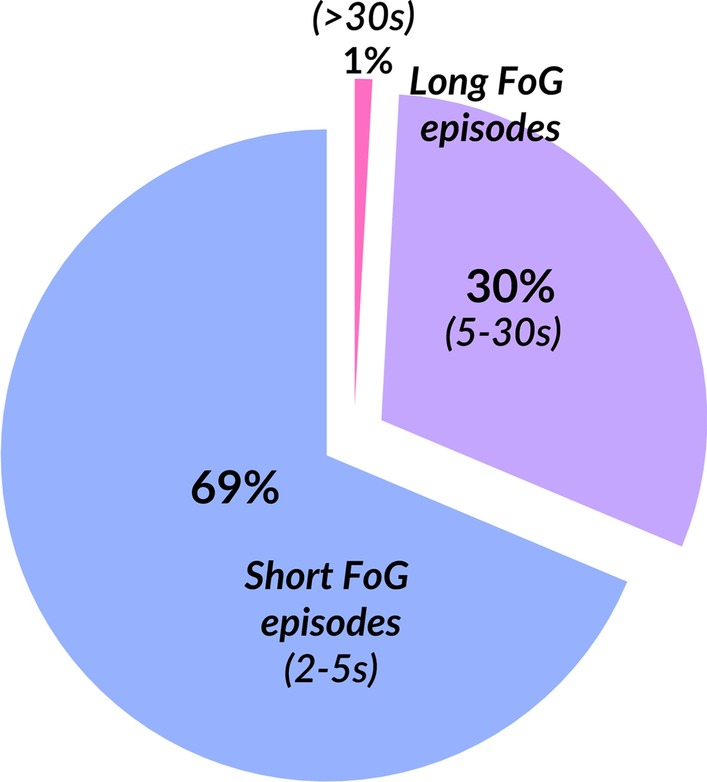
Pie chart summarizing the composition of the average FoG episodes duration

In PD + FoG, we found on average 18 FoG episodes (average of 30-min windows) where 69% of the episodes consisted of short FoG episodes (2-5 s). The remainer were long episodes (> 5), and only 1% of the episodes were over 30 s, see Fig. 4.

Quantity of mobility, such as average number of turns, average number of bouts and average bout durations, were similar in people with and without FoG (Table 4). However, measures of quality of mobility, such as average pitch angle of the feet while walking, average turn angle, and average turn duration were significantly smaller in people with FoG compared to people without FoG (p < 0.05, see Table 4). In addition, variability of pitch angle of the feet during walking was significantly larger in people with FoG, compared to people without FoG (p < 0.05, see Table 4). Other measures of quality of walking and turning, such as gait speed, and average, turning, peak velocity were similar between people with and without FoG (p > 0.05, see Table 4). However, when considering disease duration as a covariate, only average turn angle was still significantly smaller (p < 0.0001) in people with FoG, compared to people without FoG.

**Fig. 5:**
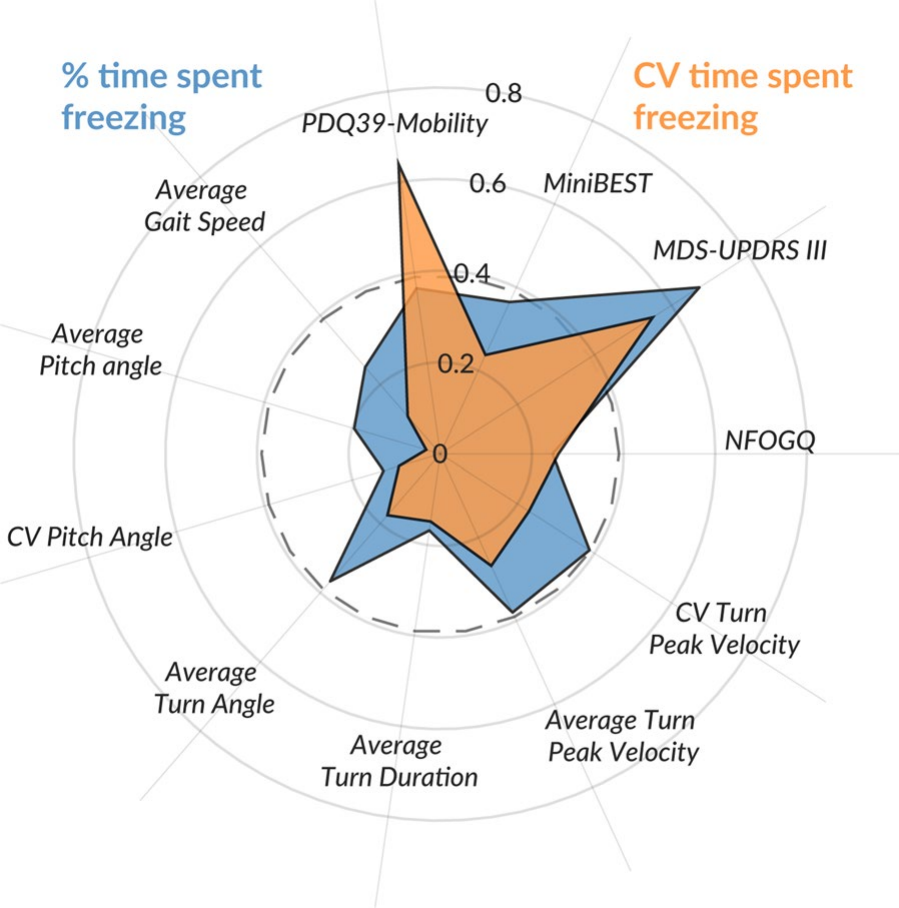
Radar plot summarizing the correlation between % of time spent FoG and its variability with clinical and objective measures of quality and quantity of walking and turning over 7 days of continuous monitoring at home

The average% of time spent freezing (in people who self-identified as freezers) was significantly associated with the MDS-UPDRS III, but not to objective measures of walking and turning at home. The CV of the time spent FoG was associated with both MDS-UPDRS Part III and to the mobility sub-score of the PDQ-39, see radar plot in Fig. 5.

## Discussion

This study introduces a novel, automated algorithm for detection and objective characterization of FoG episodes from inertial sensors on the feet. The proposed algorithm is simple and threshold-based, with one threshold based on angular velocity data and one on accelerometry data, to identify FoG episodes. Overall, we showed better agreement between clinical raters and the algorithm in detecting the number of FoG episodes in the laboratory (Study I) for long FoG episodes. In fact, for very-short and short FoG episodes, the ICCs during dual-task walking were lower compared to ICCs calculated for single-task walking.

Further, we explored this approach during unsupervised home monitoring (Study II) and found that the proposed FoG proxies, percent of walking time spent freezing and the variability of time spent freezing, were different between people with and without FoG. The percent of walking time spent freezing also was related to disease severity, measured with the MDS-UPDRS Part III and perception of mobility, measured with the mobility sub-score of the PDQ-39.

Here, we modified a threshold-based approach to detect FoG recently presented [50]. Specifically, the proposed open-source algorithm first detects periods of walking and turning [42, 50], then applies two thresholds, which need to be satisfied to label an episode as a “FoG episode”. Specifically, the first threshold is on the spectral power of the data coming from the accelerometers, a common way to identify FoG presence, based on the high-frequency components of the legs trembling [51]. This method has well-known advantages and disadvantages [24, 52], and can improve the detection of FoG episodes. To also detect FoG episodes not involving trembling of the knees, we added a threshold, based on the correlation between the right and left angular velocity of the feet. Usually, during regular walking, the correlation between right and left foot angular velocity is high, while it drops significantly prior to, and during, a FoG episode [45, 46]. The same approach can be applied to wearable sensors placed on the feet or shins. In the present study, while evaluating the performance of this approach with the clinical raters, we found that: (1) the overall agreement between clinical and objective detection of FoG is strong when the sensors are placed on the feet, and moderate when the sensors are placed on the shins, (2) both the agreement between the two clinical raters, as well as the agreement between clinical raters and objective measures are better for the short (2–5 s) and long (> 5 s) FoG duration, whereas both agreements are poor for the very short FoG episodes (< 1 s); (3) in general the agreement in the dual-task walking condition seem lower compared to the single-task walking conditions for the short FoG episodes.

Recently, machine learning based methods [24] (neural networks, decision trees, random forest, and support vector machine) have been proposed to surpass the FoG detection abilities of threshold-based methods. However, it is still unclear whether an algorithm that matches perfectly with clinical judgement is needed, even more so, when there is still discrepancy among clinical raters with more or less experience in detecting the same FoG episodes specifically for very short FoG episodes where the agreement between different raters is poor. Moreover, despite the higher sensitivity in detecting the occurrence of even shorter FOG episodes compared to the previous method [24] (an accuracy above 90% was achieved), these approaches may require a higher computational cost, requiring up to several seconds from the occurrence of the episode to its detection, making those algorithms not suitable for real-time interventions, such as cueing. However, nowadays, the use of floating-point unit microcontrollers could overcome this limitation, in fact, such microcontrollers could compute advanced machine learning algorithms in real time with low power consumption.

Overall, the proposed approach reached AUC of 0.89 to 0.93 in discriminating people experiencing FoG or not, when FoG was classified by movement disorder neurologists. These AUCs and relative sensitivity, specificity and accuracy are similar to what reported in the literature using a variety of approaches [23, 24]. Lastly, we observed lower agreement between raters and the algorithm for short episodes during dual-task walking. Although this should be verified in a separate cohort, a pontential explanation could be related to decrease smoothness of walking in people with PD and particularly in freezers [53, 54]. It could be possible that dual-task further decreases smoothness of gait, such decrease may be picked up as freezing by the algorithm but not by the clinical raters.

After comparing the algorithm with clinical judgment in the laboratory, we extended our approach to unsupervised monitoring during daily life for 7 days in 48 people with PD, 23 of which reported having FoG according to the NFOGQ. The percentage of time spent freezing was significantly higher in those people who report themselves as freezers compared to non-freezers, while the variability of time spent freezing was lower in those reporting FoG. The lower variability found in freezers may indicate that a certain amount of FoG is present across the day and week. Instead, the higher variability found in the non-freezers may either indicate that we are picking up subtle hesitations that are not constantly present over the day, therefore increasing the variability, or the high variability could be due to a high false positive rate. Therefore, to confirm this finding, we would need to first validate the algorithm during daily life and follow longitudinally the same cohort of people with PD who does not report FoG at baseline.

Interestingly, we found that the short, 2–5 s duration, FoG episodes account for 69% of all episodes, while the rest are long (5–30 s) episodes, 1% of which have duration over 30 s. This suggests that short FoG episodes are the most common during the day. The percent of walking time spent freezing and its variability were related to disease severity, measured with the MDS-UPDRS Part III and to perceived mobility, measured with the PDQ-39, only in the freezers. The association between percent time spent freezing and the MDS-UPDRS Part III is not totally surprising, as freezing severity tends to increase with disease severity. The association between the variability of time spent freezing and mobility perception suggests that FoG may affect the perception of mobility in people with PD. Our objective measures of FoG during 7 days of continuous monitoring were not significantly associated with the NFOGQ performed at the beginning of the study, indicating that perception of FoG may be differ from the measured FoG over a week of monitoring. Although surprising, this may, in part, be explained by the items composing the NFOGQ. The questionnaire asks about the impact of freezing in daily life, in addition to the presence and severity of freezing. For some people, even mild freezing may significantly disturb walking,and cause fear of falling that may significantly impact or cause people to avoid activities of daily life.

We also characterize quantity and quality of walking and turning over 7 days of continuous monitoring. Our findings are in keeping with studies showing that *quantity* of walking and turning is similar among people with and without FoG [42, 55] while *quality* of walking and turning may be more affected in people with FoG. Specifically, the average pitch angle at initial contact of foot with the ground was significantly smaller in freezers compared to non-freezers, consistent with more shuffling gait and more falls in freezers than non-freezers. The large variability of the pitch angle at initial contact and the high variability of the time spent freezing could potentially reflect fluctuations in number of freezing episodes due to periodic medication intake throughout the day.

In addition, the average turning angle was smaller in freezers compared to non-freezers, as previously reported in a larger cohort [42]; and such difference could potentially be attributed to the fact that freezers may avoid larger turning angles, known to elicit more freezing, and explain that turning duration was significantly shorter in freezers. However, after correcting these outcomes for disease duration, only turning angle was still statistically significant, suggesting that gait disturbances such as shuffling are related to disease duration more than to freezing of gait. Turning angle was still significantly smaller in freezers compared to non-freezers after correcting for disease duration suggesting that freezers may modify their turning in order to avoid FoG. However, it is also possible that average turning angles were measured as small in freezers because they hesitated during a turn such that a large turn was detected as several small turns.

These findings, although promising, should be taken cautiously. Future work will need to validate the algorithm on a new dataset, increase the number of subjects at home and determine the validity of our objective freezing measures in daily life. Specifically, we plan to use either a mini-camera pointed at the feet or pressure insoles as a gold standard comparison for home recording of gait and turning, for comparison with the inertial sensor data. It is also possible that some participants with FoG show akinetic freezing, not involving trembling of the knees, so these events may not be identified with our threshold approach. At this time, we hope that by making this algorithm available to researchers, we could, together, further improve FoG detection.

## Conclusions

Overall, here we presented an objective measure of freezing with wearable technology to be used in the laboratory. Its validity would need to be determined for continuous monitoring in unsupervised settings. These metrics could have tremendous value to assess the efficacy of interventions such as medications and rehabilitation on the quality of mobility and frequency of FoG during community-living.

## Figures and Tables

**Table 5:**
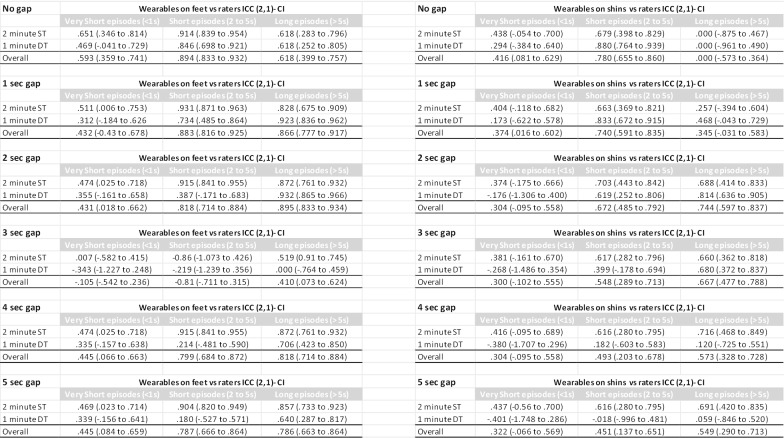
ICCs changes based on merging or not subsequent FoG episodes. Window of merging goes from no merging at all (No gap) to 5s gap in merging subsequent episodes. The ICC is between the average of the clinical raters and the algorithm based on IMUs on the feet and the algorithm based on IMUs on the shins.

## Data Availability

The algorithm generated is available in the https://github.com/BDLab-OR/FoGdetection. repository with few data as example. The full datasets used and/or analysed during the current study are available from the corresponding author on reasonable request.

## Appendix

See Table 5.

## Abbreviations

PD: Parkinson’s disease
FoG: Freezing of Gait
NFOGQ: New Freezing of Gait Questionnaire
PDQ39: Parkinson’s Disease Questionnaire
MDS-UPDRS: Movement Disorders Society Unified Parkinson’s Disease Rating Scale
FFT: Fast fourier transform
ICC: Intra-class correlation coefficient
CV: Coefficient of variation
PD-FoG: Participants with PD without freezing of gait
PD + FoG: Participants with PD with freezing of gait
AUC: Area under the ROC curve

## References

[1] Morris ME, Huxham FE, McGinley J, Iansek R (2001). Gait disorders and gait rehabilitation in Parkinson's disease. Adv Neurol.

[2] Morris ME, Iansek R, Galna B (2008). Gait festination and freezing in Parkinson's disease: pathogenesis and rehabilitation. Mov Disord.

[3] Giladi N, Nieuwboer A (2008). Understanding and treating freezing of gait in parkinsonism, proposed working definition, and setting the stage. Mov Disord.

[4] Nutt JG, Bloem BR, Giladi N, Hallett M, Horak FB, Nieuwboer A (2011). Freezing of gait: moving forward on a mysterious clinical phenomenon. Lancet Neurol.

[5] Moore O, Peretz C, Giladi N (2007). Freezing of gait affects quality of life of peoples with Parkinson's disease beyond its relationships with mobility and gait. Mov Disord.

[6] Okuma Y (2014). Practical approach to freezing of gait in Parkinson's disease. Pract Neurol.

[7] Nonnekes J, Snijders AH, Nutt JG, Deuschl G, Giladi N, Bloem BR (2015). Freezing of gait: a practical approach to management. Lancet Neurol.

[8] Mancini M, Bloem BR, Horak FB, Lewis SJG, Nieuwboer A, Nonnekes J (2019). Clinical and methodological challenges for assessing freezing of gait: future perspectives. Mov Disord.

[9] Morris TR, Cho C, Dilda V, Shine JM, Naismith SL, Lewis SJ (2012). A comparison of clinical and objective measures of freezing of gait in Parkinson's disease. Parkinsonism Relat Disord.

[10] Gilat M (2019). How to annotate freezing of gait from video: a standardized method using open-source software. J Parkinson's Dis.

[11] Morris TR, Cho C, Dilda V, Shine JM, Naismith SL, Lewis SJ (2013). Clinical assessment of freezing of gait in Parkinson's disease from computer-generated animation. Gait Posture.

[12] Rahman S, Griffin HJ, Quinn NP, Jahanshahi M (2008). The factors that induce or overcome freezing of gait in parkinson’s disease. Behav Neurol.

[13] Snijders AH, Nijkrake MJ, Bakker M, Munneke M, Wind C, Bloem BR (2008). Clinimetrics of freezing of gait. Mov Disord.

[14] Spildooren J, Vercruysse S, Desloovere K, Vandenberghe W, Kerckhofs E, Nieuwboer A (2010). Freezing of gait in Parkinson's disease: the impact of dual-tasking and turning. Mov Disord.

[15] Plotnik M, Giladi N, Hausdorff JM (2009). Bilateral coordination of gait and Parkinson’s disease: the effects of dual tasking. J Neurol Neurosurg Psychiatry.

[16] Robles-García V, Corral-Bergantiños Y, Espinosa N, Jácome MA, García-Sancho C, Cudeiro J (2015). spatiotemporal gait patterns during overt and covert evaluation in patients with Parkinson´s disease and healthy subjects: is there a hawthorne effect?. J Appl Biomech.

[17] Heremans E, Nieuwboer A, Vercruysse S (2013). Freezing of gait in Parkinson's disease: where are we now?. Curr Neurol Neurosci Rep.

[18] Snijders AH, Haaxma CA, Hagen YJ, Munneke M, Bloem BR (2012). Freezer or non-freezer: clinical assessment of freezing of gait. Parkinsonism Relat Disord.

[19] Giladi N, Hausdorff JM (2006). The role of mental function in the pathogenesis of freezing of gait in Parkinson's disease. J Neurol Sci.

[20] Del Din S, Godfrey A, Mazza C, Lord S, Rochester L (2016). Free-living monitoring of Parkinson's disease: Lessons from the field. Mov Disord.

[21] Patel S, Park H, Bonato P, Chan L, Rodgers M (2012). A review of wearable sensors and systems with application in rehabilitation. J Neuroeng Rehabil.

[22] Thorp JE, Adamczyk PG, Ploeg HL, Pickett KA (2018). Monitoring motor symptoms during activities of daily living in individuals with Parkinson's disease. Front Neurol.

[23] Silva de Lima AL, Evers LJW, Hahn T, Bataille L, Hamilton JL, Little MA (2017). Freezing of gait and fall detection in Parkinson's disease using wearable sensors: a systematic review. J Neurol.

[24] Pardoel S, Kofman J, Nantel J, Lemaire ED (2019). Wearable-sensor-based detection and prediction of freezing of gait in Parkinson's disease: a review. Sensors.

[25] Sigcha L, Costa N, Pavón I, Costa S, Arezes P, López JM (2020). Deep learning approaches for detecting freezing of gait in Parkinson's disease patients through on-body acceleration sensors. Sensors.

[26] Rodríguez-Martín D, Samà A, Pérez-López C, Català A, Moreno Arostegui JM, Cabestany J (2017). Home detection of freezing of gait using support vector machines through a single waist-worn triaxial accelerometer. PLoS ONE.

[27] Rodríguez-Martín D, Samà Monsonís A, Pérez C, Català A, Mestre B, Alcaine S, et al. Comparison of Features, Window Sizes and Classifiers in Detecting Freezing of Gait in Patients with Parkinson's Disease Through a Waist-Worn Accelerometer. 2016.

[28] Tzallas AT, Tsipouras MG, Rigas G, Tsalikakis DG, Karvounis EC, Chondrogiorgi M (2014). PERFORM: a system for monitoring, assessment and management of patients with Parkinson's disease. Sensors (Basel, Switzerland).

[29] Camps J, Samà A, Martín M, Rodríguez-Martín D, Pérez-López C, Alcaine S (2017). Deep learning for detecting freezing of gait episodes in Parkinson’s disease based on accelerometers.

[30] Mazilu S, Calatroni A, Gazit E, Roggen D, Hausdorff JM, Tröster G (2013). Feature learning for detection and prediction of freezing of gait in Parkinson’s disease.

[31] Mazilu S, Hardegger M, Zhu Z, Roggen D, Tröster G, Plotnik M, et al. editors. Online detection of freezing of gait with smartphones and machine learning techniques. 2012 6th International Conference on Pervasive Computing Technologies for Healthcare (PervasiveHealth) and Workshops; 2012 21–24 May 2012.

[32] Samà A, Rodríguez-Martín D, Pérez-López C, Català A, Alcaine S, Mestre B (2018). Determining the optimal features in freezing of gait detection through a single waist accelerometer in home environments. Pattern Recog Lett.

[33] Nieuwboer A, Rochester L, Herman T, Vandenberghe W, Emil GE, Thomaes T (2009). Reliability of the new freezing of gait questionnaire: agreement between patients with Parkinson's disease and their carers. Gait Posture.

[34] Giladi N (2008). Medical treatment of freezing of gait. Mov Disord.

[35] Mancini M, Smulders K, Harker G, Stuart S, Nutt JG (2018). Assessment of the ability of open- and closed-loop cueing to improve turning and freezing in people with Parkinson's disease. Sci Rep.

[36] Goetz CG (2010). Movement disorder society-unified Parkinson's disease rating scale (MDS-UPDRS): a new scale for the evaluation of Parkinson's disease. Rev Neurol (Paris).

[37] Shah VV, McNames J, Mancini M, Carlson-Kuhta P, Spain RI, Nutt JG (2020). Quantity and quality of gait and turning in people with multiple sclerosis, Parkinson's disease and matched controls during daily living. J Neurol.

[38] Jenkinson C, Fitzpatrick R, Peto V, Greenhall R, Hyman N (1997). The Parkinson's Disease Questionnaire (PDQ-39): development and validation of a Parkinson's disease summary index score. Age Ageing.

[39] El-Gohary M, Pearson S, McNames J, Mancini M, Horak F, Mellone S (2013). Continuous monitoring of turning in patients with movement disability. Sensors.

[40] Mancini M, El-Gohary M, Pearson S, McNames J, Schlueter H, Nutt JG (2015). Continuous monitoring of turning in Parkinson’s disease: rehabilitation potential. Neurorehabilitation.

[41] Mancini M, Schlueter H, El-Gohary M, Mattek N, Duncan C, Kaye J (2016). Continuous monitoring of turning mobility and its association to falls and cognitive function: a pilot study. J Gerontol Ser A.

[42] Mancini M, Weiss A, Herman T, Hausdorff JM (2018). Turn around freezing: community-living turning behavior in people with Parkinson’s disease. Front Neurol.

[43] Moore ST, Yungher DA, Morris TR, Dilda V, MacDougall HG, Shine JM (2013). Autonomous identification of freezing of gait in Parkinson's disease from lower-body segmental accelerometry. J Neuroeng Rehabil.

[44] Mancini M, Smulders K, Cohen RG, Horak FB, Giladi N, Nutt JG (2017). The clinical significance of freezing while turning in Parkinson's disease. Neuroscience.

[45] Palmerini L, Rocchi L, Mazilu S, Gazit E, Hausdorff JM, Chiari L (2017). Identification of characteristic motor patterns preceding freezing of gait in Parkinson's disease using wearable sensors. Front Neurol.

[46] Dale ML, Mancini M, Curtze C, Horak FB, Fling BW (2016). Freezing of gait associated with a corpus callosum lesion. J Clin Mov Disord.

[47] Shah V, McNames J, Mancini M, Carlson-Kuhta P, El-Gohary M, Nutt J (2020). Digital biomarkers of mobility in Parkinson's disease during daily living. J Parkinson’s Dis.

[48] Shrout PE, Fleiss JL (1979). Intraclass correlations: uses in assessing rater reliability. Psychol Bull.

[49] Schaafsma JD, Balash Y, Gurevich T, Bartels AL, Hausdorff JM, Giladi N (2003). Characterization of freezing of gait subtypes and the response of each to levodopa in Parkinson's disease. Eur J Neurol.

[50] Mancini M, Curtze C, Stuart S, El-Gohary M, James, McNames, et al. The Impact Of Freezing Of Gait On Balance Perception And Mobility In Community-Living With Parkinson'S Disease. Conference proceedings : Annual International Conference of the IEEE Engineering in Medicine and Biology Society IEEE Engineering in Medicine and Biology Society Annual Conference. 2018;2018:3040–3043. 10.1109/EMBC.2018.8512910 PMC7765340 30441036

[51] Moore ST, MacDougall HG, Ondo WG (2008). Ambulatory monitoring of freezing of gait in Parkinson's disease. J Neurosci Methods.

[52] Bachlin M, Plotnik M, Roggen D, Maidan I, Hausdorff JM, Giladi N (2010). Wearable assistant for Parkinson's disease patients with the freezing of gait symptom. IEEE Trans Inf Technol Biomed.

[53] Beck Y, Herman T, Brozgol M, Giladi N, Mirelman A, Hausdorff JM (2018). SPARC: a new approach to quantifying gait smoothness in patients with Parkinson's disease. J Neuroeng Rehabil.

[54] Pinto C, Schuch CP, Balbinot G, Salazar AP, Hennig EM, Kleiner AFR (2019). Movement smoothness during a functional mobility task in subjects with Parkinson’s disease and freezing of gait – an analysis using inertial measurement units. J Neuroeng Rehabil.

[55] Weiss A, Herman T, Giladi N, Hausdorff JM (2015). New evidence for gait abnormalities among Parkinson's disease patients who suffer from freezing of gait: insights using a body-fixed sensor worn for 3 days. J Neural Transmission.

